# The COMT Val158 allele is associated with impaired delayed-match-to-sample performance in ADHD

**DOI:** 10.1186/1744-9081-8-25

**Published:** 2012-05-28

**Authors:** Natasha Matthews, Alasdair Vance, Tarrant D R Cummins, Joseph Wagner, Amanda Connolly, Jacqueline Yamada, Paul J Lockhart, Ajay Panwar, Robyn H Wallace, Mark A Bellgrove

**Affiliations:** 1 The University of Queensland, Queensland Brain Institute and School of Psychology, St Lucia, 4072, Brisbane, Australia; 2 Academic Child Psychiatry Unit, Department of Paediatrics, University of Melbourne, Royal Children's Hospital, Parkville, Australia; 3 Bruce Lefroy Centre, Murdoch Children’s Research Institute, Parkville, Australia; 4 The University of Queensland, Queensland Brain Institute, Brisbane, Australia; 5 The University of Queensland, School of Chemistry and Molecular Biosciences, Brisbane, Australia

**Keywords:** Attention-deficit-hyperactivity-disorder, Working memory, COMT

## Abstract

**Background:**

This study explored the association between three measures of working memory ability and genetic variation in a range of catecholamine genes in a sample of children with ADHD.

**Methods:**

One hundred and eighteen children with ADHD performed three working memory measures taken from the CANTAB battery (Spatial Span, Delayed-match-to-sample, and Spatial Working Memory). Associations between performance on working memory measures and allelic variation in catecholamine genes (including those for the noradrenaline transporter [NET1], the dopamine D4 and D2 receptor genes [DRD4; DRD2], the gene encoding dopamine beta hydroxylase [DBH] and catechol-O-methyl transferase [COMT]) were investigated using regression models that controlled for age, IQ, gender and medication status on the day of test.

**Results:**

Significant associations were found between performance on the delayed-match-to-sample task and COMT genotype. More specifically, *val/val* homozygotes produced significantly more errors than did children who carried a least one *met* allele. There were no further associations between allelic variants and performance across the other working memory tasks.

**Conclusions:**

The working memory measures employed in the present study differed in the degree to which accurate task performance depended upon either the dynamic updating and/or manipulation of items in working memory, as in the spatial span and spatial working memory tasks, or upon the stable maintenance of representations, as in the delay-match–to-sample task. The results are interpreted as evidence of a relationship between tonic dopamine levels associated with the *met* COMT allele and the maintenance of stable working memory representations required to perform the delayed-match-to-sample-task.

## Background

Attention-deficit/hyperactivity disorder (ADHD) is a common neuropsychiatric disorder, characterized by age-inappropriate symptoms of inattention, motor over-activity and impulsiveness, observed before the age of seven [1]. The disorder has an estimated prevalence of 3-8% in school-aged children [2] and causes significant lifetime academic, social and occupational impairment [3]. Family, twin and adoption studies suggest a significant genetic contribution to ADHD, with heritability estimates between 70-90% [4]. Despite this strong genetic loading mapping specific genes has proven difficult, in part due to the heterogeneous clinical presentation of ADHD. It has recently been proposed that cognitive endophenotypes, such as working memory ability, may increase the ability to detect subtle genetic effects by providing an index of neurobiological processes that are more closely related to the products of gene expression than diagnostic categories [5].

Working memory enables the temporary maintenance, updating and manipulation of relevant information in a limited capacity cognitive system and has a significant genetic component, with heritability estimates ranging from 33-49% [6]. Working memory processes have long been implicated in theoretical models of ADHD and a growing literature confirms the robust nature of working memory deficits in the disorder [7].

Given that working memory ability permits internal representations of information to guide decision-making and behaviour, impairments in working memory are likely to have important functional consequences for many higher-order processes including language, planning and goal-directed behaviour [8]. Consequently, measures of impaired executive function, including working memory, have been linked to increased risk for grade retention and decreased academic achievement in children with ADHD [9]. Understanding the genetic determinants of individual differences in working memory ability in children with ADHD may therefore have important prognostic value.

Working memory is sub-served by a broad neural network involving the ventrolateral and dorsolateral prefrontal cortices, the striatum and the inferior parietal lobes [10,11]. However, within this network there is mounting evidence for a critical role for the prefrontal cortex, particularly for visuospatial working memory [12]. Within the prefrontal cortex spatial working memory ability is strongly mediated by the catecholamines dopamine and noradrenaline with both displaying an inverted U-shaped function whereby either too much or too little of either will result in sub-optimal working memory performance [13,14].

Given the sensitivity of working memory ability to catecholamine levels, genes affecting these systems in prefrontal cortex may have utility for explaining individual differences in working memory ability. One of the most widely studied genes of relevance to prefrontal cognition is the catechol-O-methyltransferase (COMT) gene. COMT produces an enzyme that breaks down catecholamines, thus clearing them from the synaptic cleft. COMT is the primary mechanism of dopamine clearance in prefrontal cortex [15], in part because other regulators of synaptic dopamine, such as the dopamine transporter (DAT) are sparse in this region. A single nucleotide polymorphism (SNP: known as Val158Met or rs4680) comprising a guanine (G) to adenine (A) mutation results in an amino acid substitution of methionine (*met*) for valine (*val*) in enzyme synthesis. The more thermostable *val* allele is associated with greater dopamine degradation and hence less synaptic dopamine than the less stable *met* allele [16]. This change in thermostability may have consequences for working memory ability. The *val* rather than the *met* allele has been associated with reduced prefrontal cortical activation in functional neuroimaging studies of verbal working memory in healthy adults [17], and with poorer working memory performance in both adult populations [18,19] and in healthy children [20]. It is reasonable therefore to hypothesize that working memory deficits in ADHD could be underpinned, at least in part, by the increased turnover of prefrontal dopamine that is associated with the *val* variant. Nevertheless, further examination of other genes of the catecholamine system that could also influence working memory is now also warranted.

To date, studies investigating the COMT variant as a risk factor for ADHD have been inconclusive. The majority of family-based studies that have examined the COMT polymorphism in ADHD have found no significant association, while associations with both the *met* and *val* alleles have also been reported [21]. Only a few studies have specifically investigated the relationship between COMT genotype and cognitive variables in ADHD populations and the results are inconclusive [22-24].

One explanation for the lack of consistency reported in the ADHD literature might be the varied characteristics of the tasks used to assay prefrontal function. There is evidence that prefrontal cortical dopamine is particularly important in the updating and stabilization of representations in working memory [25]. We therefore hypothesized that any associations with COMT genotype would be most pronounced in tasks requiring the active maintenance of a stable representation *rather than* the dynamic updating and manipulation of items stored in working memory.

The present study explored working memory in children with ADHD across a number of tasks that differed in the demands they placed on the necessity for maintenance of a stable representation versus dynamically updating working memory representations. Moreover, here we focus on non-verbal working memory tasks as previous research has suggested larger effect sizes for spatial, as compared to verbal, working memory tasks in ADHD [7]. We also explored for the first time the relationship between working memory ability in ADHD and allelic variation across a range of catecholamine genes, including those for the noradrenaline transporter (NET1), the dopamine D4 and D2 receptor genes (DRD4; DRD2), the gene encoding dopamine beta hydroxylase (DBH) and COMT. Each of these genes has been implicated as potentially increasing genetic susceptibility to ADHD or has been related to executive function ability [26] but, there has not been a systematic investigation of their association with spatial working memory phenotypes in a large ADHD cohort.

## Materials and methods

### Participants

One hundred and eighteen Caucasian children with ADHD (6–16 years; 101 males: Table 1) were recruited through a specialized clinic for ADHD located at the Royal Children’s Hospital (RCH) in Melbourne, Australia. Participants were referred to this service by school support staff because of difficulties noted in the school classroom and/or playground. Ethics approval for the study was obtained from the Human Research Ethics Committees of the University of Queensland, and the Royal Children’s Hospital Melbourne. Informed consent was obtained from the parents of all participants and where appropriate, from the participants themselves. ADHD diagnosis was determined using the parent version of the Anxiety Disorders Interview Schedule for Children (A-DISC [27]) according to DSM-IV criteria [1]. Two experienced psychiatrists reviewed all clinical diagnoses. Parents were required to complete the Conners’ Parent ADHD rating Scale-Revised: Long Version (CPRS-R:L [28]), and children with ADHD were required to have a Global Index T-score > 65 for inclusion in the study (Table 1), symptom pervasiveness was established using teacher responses on the Teacher Report Form (TRF [29]). Ninety-two (78%) children with ADHD met criteria for ADHD-combined type, eighteen (15.3%) for inattentive type, and eight (6.8%) for the hyperactive-impulsive type. Children were excluded if they had a co-morbid diagnosis of major depressive disorder. Sixty-six (55.9%) of the participants met criteria for co-morbid conduct disorder and 28 (23.7%) met criteria for oppositional defiant disorder. Participant IQ was assessed by the WISC-IV [30] (Table 1) and participants were excluded if they had an estimated full-scale IQ≤70, or if they had previously been diagnosed with impaired sensorimotor skills (Scored Developmental Neurological Examination [31]) or learning disabilities (WRAT 3 [32]).

**Table 1:** **Demographic and clinical data for children with ADHD (*n*= 118)**

**Measure**	**Mean (SD)**
Age	9.7 years (2.6)
Full scale IQ	91.4 (11.2)
Conners’ ADHD Index score	74.2 (9.9)
Conners’ Global Index Total score	79.9 (6.9)
Conners’ *DSM-IV* Inattentive score	72.6 (8.4)
Conners’ *DSM-IV* Hyperactive/Impulsive score	81.4 (8.5)
Conners’ *DSM-IV* Total score	79.2 (8.4)

Ninety-five (80.5%) participants were medication free at the time of testing, including seventy-four (62.7%) participants who were medication naïve and twenty-one (17.7%) who underwent a medication wash-out period of at least 24 h prior to the testing session. The remaining twenty-three (19.4%) participants were on active medication at the time of testing.

### Stimuli and procedure

Participants performed three measures of working memory from the Cambridge Neuropsychological Test Automated Battery (CANTAB): spatial working memory (SWM), spatial span (SP), and delayed-match-to-sample (DMTS). All CANTAB measures were presented on a high-resolution IBM colour monitor with a touch sensitive screen at a viewing distance of 0.5 meters. The CANTAB is a computerized battery of neuropsychological tests originally developed for use with normal and neurologically-impaired populations between the ages of four and ninety, and which has also been successfully applied to children and adolescents with ADHD [33]. The CANTAB working memory measures have been shown to have a substantial heritable component [34], suggesting that they are suitable phenotypes for genetic analysis.

#### Spatial span

Participants were presented with 10 boxes that served as spatial placeholders. On each trial a subset of the boxes flashed sequentially. Participants were required to reproduce the spatial sequence by touching the boxes on the screen in the order in which they were presented. The length of the test sequence was increased by one item contingent upon performance, up to a maximum of nine items. Maximum correct sequence length was defined as their spatial memory span.

#### Spatial working memory

On each trial of this task participants were presented with a number of coloured squares located at different spatial locations. In order to find a token, participants were required to engage in a self-guided search of the coloured squares by touching each square in turn to identify if it concealed a token. Each square contained only one token on a given trial. Returning to a location in which a token had already been found on a given trial was scored as a search error. The number of squares presented on each trial (the display set size) was increased throughout the task: 3, 4, 6 to 8 items. Search error score was calculated for each set size and summed to produce a total error score.

A strategy score was also defined as a measure of the consistency with which a search strategy was employed; this is estimated from the number of searches that start with the same location within each of the six-item and eight-item searches. A high score indicates low use of strategy.

#### Delayed match to sample

Participants were shown a complex visual pattern and were then required to select the identical pattern from among four possible response alternatives. The difficulty of the task was manipulated by varying the delay between the presentation of the test stimulus and the presentation of the four response stimuli (0, 4, to 12 s). A simultaneous matching condition was also included to control for perceptual deficits. Performance was defined as the percentage of correct responses.

### Genotyping

Genotyping on saliva samples provided by participants was performed on ten single nucleotide polymorphisms (SNPs) in the genes for dopamine beta hydroxylase (DBH: rs1611115, rs2519152 [35-37]), the dopamine D2 receptor (DRD2: rs1800497, rs6277, rs1079596, rs2075654 [38]), DRD4 (rs1800955 [39]), the noradrenaline transporter (SLC6A2 or NET1: rs3785155, rs880711 [38]) and catechol-O-methyltransferase (COMT: rs4680 [24]). These markers were chosen based upon prior evidence of the SNP conferring risk to ADHD or to neurocognitive deficits in ADHD or upon functional evidence for the SNP and its theoretical link to working memory.

Genotyping of all SNPs was performed by the Australian Genome Research Facility (AGRF) using iPLEX GOLD chemistry with a Sequenom MassArray on an Autoflex Spectrometer. Genotyping failures were in the region of 4-6% across the SNPs investigated.

### Genetic association analysis

Permutation methods are considered the gold standard for multiple comparison correction because they provide unbiased type 1 error control while maintaining statistical power. Accordingly we used a single step permutation method to test for genetic associations with our task variables (Please see [40-42] for a full description of single step permutation methods). Briefly, the analysis described below was performed separately for each of the working memory variables for each task (Delayed-match-to-sample, span, and spatial working memory) using Matlab (v. 2008a; http://www.mathworks.com/products/). For each genetic marker an association analysis between the task variable and genotype was performed using single-step additive, dominant, and recessive regression models that included age, gender, IQ, and medication status on the day of testing, as covariates. The absolute (unstandardised) beta value for the task variable was recorded (these values are hereafter referred to as the unshuffled test statistics). This was then followed by a single step permutation method in which each individual’s index (the profile that is made up of their score on the task variable and their scores on all of the covariates) was shuffled multiple times relative to the genetic data. For each shuffled configuration of the data, an association analysis (as described above) was performed for every genetic marker and the maximal absolute value observed for the test statistic (beta) of the task variable across all genetic markers was recorded. This process was repeated 100,000 times and a list of the maximal beta values (one beta value per shuffle) was generated. The single-step permuted p-value for any given marker was then calculated as the fraction of maximal beta values that were greater than or equal to the absolute value of the unshuffled test statistic for the marker in question. The critical p value for all analyses was 0.05/9 or .0056 (i.e., the single step corrected permutation value corrected for the number of tasks [3] and the number of genetic models [3]).

Genotype frequencies for each marker are presented in Table 2. The results of the association analysis for each working memory phenotype are presented in Table 3 and in Additional file 1: Tables S1–S3. All markers were in Hardy-Weinberg equilibrium.

**Table 2:** Genotype frequencies for the 10 SNPs investigated

**Gene**	**Polymorphism**	**Allele minor/major**	**Genotype count (percentage)**		**Minor allele frequency**
NET	rs880711	A/G	A/A	4 (3.5)	0.18
			G/A	32 (28.3)	
			G/G	77 (68.1)	
	rs3785155	A/G	A/A	2 (1.8)	0.14
			A/G	28 (25)	
			G/G	82 (73.2)	
DRD2	rs1079596	T/C	T/T	2 (1.8)	0.15
			C/T	29 (25.7)	
			C/C	82 (72.6)	
	rs1800497	A/G	A/A	4 (3.5)	0.22
			A/G	42 (37.2)	
			G/G	67 (59.3)	
	rs2075654	T/C	T/T	2 (1.8)	0.15
			T/C	29 (25.7)	
			C/C	82 (72.6)	
	rs6277	G/A	G/G	24 (21.2)	0.47
			A/G	58 (51.3)	
			A/A	31 (27.4)	
DBH	rs1611115	T/C	T/T	9 (8.0)	0.27
			T/C	43 (38.1)	
			C/C	61 (54)	
	rs2519152	C/T	C/C	25 (22.3)	0.44
			T/C	49 (43.8)	
			T/T	38 (33.9)	
DRD4	rs1800955	C/T	C/C	20 (18)	0.46
			T/C	62 (55.9)	
			T/T	29 (26.1)	
COMT*	rs4680	G/A	G/G	25 (22.1)	0.44
			G/A	49 (43.3)	
			A/A	39 (34.5)	

* For the COMT SNP ‘G’ refers to the ‘Val’ allele and ‘A’ refers to the ‘Met’ allele

**Table 3:** The influence of common genetic variations on percentage correct for the DMTS task

**Gene**	**Polymorphism**	**Model**	**p-value (obtained)**	**p-value (corrected)**
NET	rs880711	Dominant	0.526	0.997
		Recessive	0.047	0.868
		Additive	0.868	1.000
	rs3785155	Dominant	0.504	0.997
		Recessive	0.002	0.824
		Additive	0.833	1.000
DRD2	rs1079596	Dominant	0.309	0.952
		Recessive	0.975	1.000
		Additive	0.361	0.993
	rs1800497	Dominant	0.609	0.999
		Recessive	0.861	1.000
		Additive	0.615	1.000
	rs2075654	Dominant	0.309	0.952
		Recessive	0.975	1.000
		Additive	0.361	0.993
	rs6277	Dominant	0.695	1.000
		Recessive	0.716	0.999
		Additive	0.642	0.999
DBH	rs1611115	Dominant	0.249	0.850
		Recessive	0.825	1.000
		Additive	0.325	0.945
	rs2519152	Dominant	0.915	1.000
		Recessive	0.322	0.813
		Additive	0.536	1.000
DRD4	rs1800955	Dominant	0.664	1.000
		Recessive	0.720	1.000
		Additive	0.612	1.000
COMT	rs4680	Dominant	0.291	0.921
		**Recessive**	**0.00078**	**0.0033**
		Additive	0.012	0.039

## Results

### Working memory phenotypes

#### Spatial span

Children with ADHD had a working memory span of approximately 5 items on the SP task (*M* = 4.7, *SD* = 1.4). There were no significant associations between SP and genotype (Additional file 1: Table S1).

#### Spatial working memory

The average error rate for SWM across all display set sizes in the ADHD children was 50.35 (*SD* = 19.1). There was a significant main effect of display set size *F*(3,351) = 660.85, *p* < 0.01, and post hoc analysis confirmed that this was due to a significant increase in search errors with increasing display set size (*p’*s <0.05). The mean strategy score for the ADHD group was 37.51 (*SD* = 3.23).

There were no significant associations between any of the markers and total errors or strategy score (Additional file 1: Table S2).

#### Delayed matched to sample

The children with ADHD had correct responses of 87.95% (*SD* = 14.17) on the simultaneous condition. A repeated measures ANOVA conducted on the delay conditions confirmed that there was a significant main effect of delay *F*(2,232) = 33.43, *p* <0.001, with a significant difference in mean percentage correct responses between the 12 and both the 4 and 0 s delay conditions (*p’s* < 0.05).

Association analyses were performed using the percent correct on the simultaneous condition and the percent correct averaged across all delay conditions (*M* = 61.51%, *SD* = 16.69). There were no significant associations between any of the markers and performance on the simultaneous condition (Additional file 1: Table S3).

**Figure 1:**
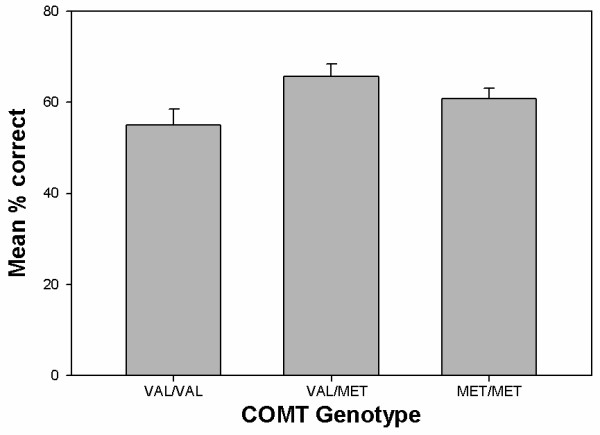
**Mean percentage correct performance averaged across 0, 4, and 12 s delay conditions in the delayed-match-to-sample task (adjusted for age, IQ, gender, and medication status) as a function of COMT genotype.** Error bars represent standard error of the mean.

Percentage of correct responses across delay conditions was significantly associated with COMT genotype under a recessive model at the corrected level (*p* = 0.0033) (Table 3). As shown in Figure 1 individuals with the *val/val* genotype had the lowest accuracies overall (*M* = 51.67%, *SD* = 15.56), with *val/met* (*M* = 65.67%, *SD* = 17.67) and *met/met* (*M* = 60.67%, *SD* = 14.83) individuals performing similarly.

## Discussion

A robust literature demonstrates that working memory ability is impaired in individuals with ADHD [7]. Working memory is known to be reliant upon prefrontal catecholamine levels [14], with COMT being an important regulator of this system [15]. Here, for the first time in children with ADHD, we explored the relationship between allelic variation in a broad set of catecholamine genes, including COMT, and measures of working memory.

In children with ADHD there was a significant relationship between COMT genotype and performance on the DMTS task. *Val/val* homozygotes had lower percent correct scores compared to those who carried at least one *met* allele. There was no relationship between COMT genotype and any of the other working memory measures investigated. Performance on a working memory task requires the co-ordination of a number of abilities: information must be accurately encoded into working memory, maintained and/or manipulated depending on task demands, and finally retrieved. COMT genotype may be related to one or more of these sub-processes in children with ADHD.

Bruder et al. [43] investigated the relationship between COMT genotype and performance on a number of working memory tasks in a healthy adult population. They found that COMT genotype was related to performance on a Letter-Number Sequencing Task, but not to performance on a Spatial Delayed Response task, n-back task, or Wisconsin Card Sorting Task. The authors argued that the Letter-Number Sequencing task was more taxing of executive processes and thus, that COMT genotype is more closely related to the manipulation of items in working memory than to maintenance.

The present results suggest an alternative interpretation; here there was an association between COMT and performance on the DMTS task, which relies on successful working memory maintenance, but not with the SP or SWM tasks, which place greater executive demands on the updating and manipulation of items. Within the DMTS task there was no interaction between COMT genotype and increasing working memory delay, suggesting that COMT genotype was not related to task difficulty. Similarly, there was no relationship between COMT genotype and performance on the simultaneous matching condition of the DMTS task, which provides a measure of perceptual accuracy. Therefore, it may be concluded that the relationship between COMT genotype and DMTS performance may be most sensitive to either impairments in the encoding of a stable representation into working memory or in retrieval stages.

One potential explanation for our pattern of results may be found in the tonic-phasic hypothesis of dopamine regulation [44]. According to this hypothesis dopamine action is orchestrated through the co-ordination of tonic and phasic states. The COMT *met* allele increases tonic dopamine transmission, which regulates the stability of cortical activation states. Tonic dopamine stimulation has thus been hypothesized to be important for maintaining stable representations in working memory. In contrast, phasic dopamine stimulation regulates the plasticity of activation states and is believed to be important for updating and manipulating working memory representations. Our findings are consistent with the role of the *met* allele in enhancing the stability and maintenance of representations required for performance on the DMTS task. These findings suggest that the determining factor in observing associations between neurocognitive measures and COMT genotype may depend more on the nature of the representation required to perform the task, rather than, as suggested by Bruder et al. [43], the executive load of the task *per se*.

Interestingly, both chronic and acute administration of methylphenidate, a stimulant drug that alters extracellular catecholamine levels [45] has been shown to improve performance on the CANTAB DMTS task, but not on the spatial working memory task in children with ADHD [33,46,47]. It has been proposed that the therapeutic effect of methylphenidate is mediated through the enhancement of tonic rather than phasic dopamine release [45]. Therefore the DMTS task in ADHD may show sensitivity both to treatments that alter tonic prefrontal dopamine levels and to variation in COMT genotype. Further work is needed to fully characterize the specific aspects of DMTS task performance that are related to COMT genotype. Many of the commonly used measures of working memory, such as the n-back task and the Wisconsin Card Sorting Task, confound in time both the requirement for stable and flexible representations, and the working memory sub-process (be it encoding, maintenance, or retrieval) being engaged. Selection of tasks that allow for the compartmentalization of these processes will help in disentangling the relationship between COMT genotype and neurocognitive functioning in ADHD.

The finding that impaired performance on the DMTS was associated with the *val* rather than the *met* allele in children with ADHD supports previous research showing an association between poorer performance on executive tasks and the *val* allele in both healthy adults [18,19] and children [20]. Only a few previous studies have specifically explored the relationship between COMT genotype and cognitive abilities in ADHD populations, with results conflicting. Two studies found no relationship between COMT genotype and executive function in children with ADHD [22,23], while Bellgrove et al. [24] found that the *met* rather than the *val* variant was associated with impaired sustained attention performance in children with ADHD. There are a number of participant-factors that may influence the finding of a relationship between COMT genotype and executive function in children with ADHD, including history of medication and participant age. Perhaps more importantly, given the nature of the findings of the present study, it may be that cognitive task selection is also critically important.

The current study imposed a stringent correction for multiple comparisons that accounted both for the number of tasks studied and the number of SNPs and genetic models tested. The observation that the recessive model of the *val* allele survived this correction is interesting in light of other studies that have also reported recessive effects. For example, among children with ADHD *val/val* homozygotes have impaired task oriented performance [48] and increased antisocial behaviour [49] relative to carriers of at least one *met* allele. Nevertheless, we note that a nominally significant effect of the additive model (*p* = 0.039) was also found that might survive multiple comparison testing in larger samples. We repeated our analysis in just the sub-sample of participants who were medication free on the day of test (co-varying for gender, age, and IQ). Although this analysis did not survive correction for multiple comparisons it was nonetheless nominally significant (*p* = 0.02).

Since we failed to observe a relationship between COMT genotype and performance on either the SP or SWM tasks, one may conclude that performance on these tasks is perhaps influenced by other catecholamine gene variants. Nevertheless, we also failed to find evidence of association between performance on the working memory tasks and allelic variation in any of the other catecholamine genes (NET1, DRD2, DBH, DRD4) under study. There are however, a number of methodological considerations that should be considered. First, it should be noted that the low minor allele frequency associated with a number of the catecholamine SNPs may have reduced our power to detect significant associations in the relatively small sample size under study. Second, the three working memory tests used in this study were not matched for psychometric characteristics or task difficulty. In addition, although all our analyses co-varied for the medication status of the participants, it is possible that medication-related factors may have obscured associations between the catecholamine gene variants and working memory measures. The sample of children with ADHD presented here was recruited from a hospital service that specialized in assessment of children with severe behavioural disturbances and as such they presented with a high rate of conduct disorder co-morbidity. Previous research has demonstrated that the association between COMT *val/val* genotype and ADHD is modified by coexisting extreme anti-social behaviour and conduct disturbances [49]. However, Langley et al. [50] investigated whether impaired social functioning or executive control mediated the relationship between COMT and anti-social behaviour in the context of children with ADHD. Their analysis revealed a mediating effect of social but not executive functioning, suggesting that the association between COMT genotype and working memory ability reported herein is unlikely to be mediated by co-morbid conduct disturbance.

## Conclusion

In summary, the results of the present study indicate that in children with ADHD the *val* variant of the COMT gene polymorphism is associated with impaired performance on a DMTS task that requires the stable maintenance of representations in working memory, but not with performance on working memory tasks that additionally require the dynamic updating of information. Although it remains uncertain whether the *val* allele of the COMT genotype confers susceptibility to ADHD, our results suggest that this polymorphism is associated with working memory impairments in ADHD children. The association between COMT genotype and working memory impairment may have important functional significance for ADHD, given that the latter has been reliably linked to poor educational and clinical outcomes.

## Competing interests

M.A. Bellgrove has received reimbursement from Lilly Pharmaceuticals and Jansen Cilag for conference travel expenses and for speaking at conferences unrelated to the content of this manuscript. M.A. Bellgrove is also the recipient of research funding from Lilly Pharmaceuticals for work unrelated to this manuscript. All other authors report no conflicts of interest to declare.

## Authors’ contributions

NM and TDRC performed statistical analysis. NM drafted the manuscript. AV participated in study design and in drafting the manuscript. JW, AP, and PL performed the genotyping. AM and JY performed the clinical assessments. RHW participated in the development of the study with particular contribution to the selection of genetic targets. MAB conceived of the study and helped draft the manuscript. All authors read and approved the final manuscript.

## Supplementary Material

Additional file 1 Two supplementary tables are provided which contain detailed information regarding the results of analyses described in the results section. (DOCX 24 kb) Click here for file
