# Supplementary material for: The COMT Val158 allele is associated with impaired delayed-match-to-sample performance in ADHD
Source: Behav Brain Funct. 2012 May 28;8:25. doi: 10.1186/1744-9081-8-25 (PMC3413539; doi:10.1186/1744-9081-8-25)
Supplement: Additional file 1 — Two supplementary tables are provided which contain detailed information regarding the results of analyses described in the results section. (DOCX 24 kb) [file 1744-9081-8-25-S1.docx]

| Gene | Polymorphism | Model | p-value  (obtained) | p-value  (corrected) |
| --- | --- | --- | --- | --- |
| NET | rs880711 | Dominant | 0.355 | 0.962 |
|  |  | Recessive | 0.953 | 1.000 |
|  |  | Additive | 0.423 | 0.993 |
|  | rs3785155 | Dominant | 0.219 | 0.862 |
|  |  | Recessive | 0.438 | 1.000 |
|  |  | Additive | 0.373 | 0.993 |
| DRD2 | rs1079596 | Dominant | 0.342 | 0.963 |
|  |  | Recessive | 0.356 | 1.000 |
|  |  | Additive | 0.540 | 0.999 |
|  | rs1800497 | Dominant | 0.238 | 0.811 |
|  |  | Recessive | 0.179 | 0.977 |
|  |  | Additive | 0.552 | 0.999 |
|  | rs2075654 | Dominant | 0.342 | 0.963 |
|  |  | Recessive | 0.356 | 1.000 |
|  |  | Additive | 0.540 | 0.999 |
|  | rs6277 | Dominant | 0.606 | 0.999 |
|  |  | Recessive | 0.716 | 0.999 |
|  |  | Additive | 0.587 | 0.997 |
| DBH | rs1611115 | Dominant | 0.305 | 0.915 |
|  |  | Recessive | 0.518 | 0.997 |
|  |  | Additive | 0.606 | 0.999 |
|  | rs2519152 | Dominant | 0.204 | 0.786 |
|  |  | Recessive | 0.100 | 0.317 |
|  |  | Additive | 0.088 | 0.284 |
| DRD4 | rs1800955 | Dominant | 0.816 | 1.000 |
|  |  | Recessive | 0.515 | 0.984 |
|  |  | Additive | 0.595 | 0.999 |
| COMT* | rs4680 | Dominant | 0.372 | 0.975 |
|  |  | Recessive | 0.379 | 0.879 |
|  |  | Additive | 0.929 | 1.000 |

**Supplementary Tables**

**Table 1.** The influence of common genetic variation within catecholamine genes on spatial span performance.

* For the COMT SNP ‘G’ refers to the ‘Val’ allele and ‘A’ refers to the ‘Met’ allele

**Table 2.** The influence of common genetic variations on spatial working memory total errors and strategy score.

| Gene | Polymorphism | Model | p-value  (obtained) | p-value  (corrected) |
| --- | --- | --- | --- | --- |
| Total Errors | | | | |
| NET | rs880711 | Dominant | 0.965 | 1.000 |
|  |  | Recessive | 0.711 | 1.000 |
|  |  | Additive | 0.927 | 1.000 |
|  | rs3785155 | Dominant | 0.683 | 1.000 |
|  |  | Recessive | 0.240 | 0.999 |
|  |  | Additive | 0.963 | 1.000 |
| DRD2 | rs1079596 | Dominant | 0.288 | 0.930 |
|  |  | Recessive | 0.477 | 1.000 |
|  |  | Additive | 0.441 | 0.997 |
|  | rs1800497 | Dominant | 0.152 | 0.641 |
|  |  | Recessive | 0.953 | 1.000 |
|  |  | Additive | 0.221 | 0.905 |
|  | rs2075654 | Dominant | 0.287 | 0.930 |
|  |  | Recessive | 0.477 | 1.00 |
|  |  | Additive | 0.441 | 0.997 |
|  | rs6277 | Dominant | 0.212 | 0.868 |
|  |  | Recessive | 0.577 | 0.986 |
|  |  | Additive | 0.649 | 0.999 |
| DBH | rs1611115 | Dominant | 0.217 | 0.781 |
|  |  | Recessive | 0.254 | 0.934 |
|  |  | Additive | 0.642 | 0.999 |
|  | rs2519152 | Dominant | 0.090 | 0.476 |
|  |  | Recessive | 0.050 | 0.150 |
|  |  | Additive | 0.029 | 0.088 |
| DRD4 | rs1800955 | Dominant | 0.921 | 1.000 |
|  |  | Recessive | 0.867 | 1.000 |
|  |  | Additive | 0.979 | 1.000 |
| COMT | rs4680 | Dominant | 0.395 | 0.868 |
|  |  | Recessive | 0.485 | 0.958 |
|  |  | Additive | 0.353 | 0.906 |
| Strategy score | | | | |
| NET | rs880711 | Dominant | 0.141 | 0.682 |
|  |  | Recessive | 0.072 | 0.905 |
|  |  | Additive | 0.062 | 0.570 |
|  | rs3785155 | Dominant | 0.247 | 0.904 |
|  |  | Recessive | 0.051 | 0.973 |
|  |  | Additive | 0.115 | 0.842 |
| DRD2 | rs1079596 | Dominant | 0.305 | 0.948 |
|  |  | Recessive | 0.860 | 1.000 |
|  |  | Additive | 0.328 | 0.988 |
|  | rs1800497 | Dominant | 0.074 | 0.375 |
|  |  | Recessive | 0.227 | 0.988 |
|  |  | Additive | 0.050 | 0.488 |
|  | rs2075654 | Dominant | 0.305 | 0.948 |
|  |  | Recessive | 0.860 | 1.000 |
|  |  | Additive | 0.328 | 0.988 |
|  | rs6277 | Dominant | 0.492 | 0.997 |
|  |  | Recessive | 0.935 | 1.000 |
|  |  | Additive | 0.629 | 1.000 |
| DBH | rs1611115 | Dominant | 0.928 | 1.000 |
|  |  | Recessive | 0.300 | 0.961 |
|  |  | Additive | 0.610 | 0.999 |
|  | rs2519152 | Dominant | 0.081 | 0.451 |
|  |  | Recessive | 0.223 | 0.625 |
|  |  | Additive | 0.073 | 0.257 |
| DRD4 | rs1800955 | Dominant | 0.899 | 1.000 |
|  |  | Recessive | 0.764 | 1.000 |
|  |  | Additive | 0.796 | 1.000 |
| COMT* | rs4680 | Dominant | 0.917 | 1.000 |
|  |  | Recessive | 0.701 | 0.999 |
|  |  | Additive | 0.885 | 1.000 |

* For the COMT SNP ‘G’ refers to the ‘Val’ allele and ‘A’ refers to the ‘Met’ allele

**Table 3.** The influence of common genetic variations on percent correct for the simultaneous condition of the DMTS task.

| Gene | Polymorphism | Model | p-value  (obtained) | p-value  (corrected) |
| --- | --- | --- | --- | --- |
| NET | rs880711 | Dominant | 0.338 | 0.960 |
|  |  | Recessive | 0.131 | 0.959 |
|  |  | Additive | 0.774 | 1.000 |
|  | rs3785155 | Dominant | 0.749 | 1.000 |
|  |  | Recessive | 0.107 | 1.000 |
|  |  | Additive | 0.882 | 1.000 |
| DRD2 | rs1079596 | Dominant | 0.334 | 0.966 |
|  |  | Recessive | 0.364 | 1.000 |
|  |  | Additive | 0.262 | 0.974 |
|  | rs1800497 | Dominant | 0.431 | 0.983 |
|  |  | Recessive | 0.281 | 0.993 |
|  |  | Additive | 0.295 | 0.963 |
|  | rs2075654 | Dominant | 0.334 | 0.966 |
|  |  | Recessive | 0.364 | 1.000 |
|  |  | Additive | 0.262 | 0.974 |
|  | rs6277 | Dominant | 0.124 | 0.715 |
|  |  | Recessive | 0.584 | 0.988 |
|  |  | Additive | 0.194 | 0.734 |
| DBH | rs1611115 | Dominant | 0.731 | 1.000 |
|  |  | Recessive | 0.252 | 0.939 |
|  |  | Additive | 0.451 | 0.989 |
|  | rs2519152 | Dominant | 0.478 | 0.993 |
|  |  | Recessive | 0.404 | 0.906 |
|  |  | Additive | 0.359 | 0.918 |
| DRD4 | rs1800955 | Dominant | 0.139 | 0.761 |
|  |  | Recessive | 0.536 | 0.989 |
|  |  | Additive | 0.504 | 0.996 |
| COMT* | rs4680 | Dominant | 0.226 | 0.852 |
|  |  | Recessive | 0.135 | 0.461 |
|  |  | Additive | 0.110 | 0.409 |

* For the COMT SNP ‘G’ refers to the ‘Val’ allele and ‘A’ refers to the ‘Met’ allele
